# Brackish water irrigation boosts honeysuckle (*Lonicera japonica* Thunb.)-salt tolerance by regulating sodium partitioning and potassium homeostasis: implications for coastal saline soil

**DOI:** 10.3389/fpls.2025.1655009

**Published:** 2025-12-01

**Authors:** Yu Shi, Xiaojie Wang, Kaipeng Zhang, Xuepeng Liu, Jiangbao Xia, Dongjie Zhang, Xiaoshuai Zhang, Qiqi Cao, Wenjun He

**Affiliations:** 1 Shandong Key Laboratory of Eco-Environmental Science for the Yellow River Delta, Shandong University of Aeronautics, Binzhou, China; 2 Key Laboratory of Coastal Zone Environmental Processes and Ecological Remediation, Yantai Institute of Coastal Zone Research, Chinese Academy of Sciences, Yantai, China; 3 Yellow River Delta Field Observation and Research Station of Coastal Wetland Ecosystem, Chinese Academy of Sciences, Dongying, China

**Keywords:** honeysuckle, brackish water irrigation, Yellow River Delta, coastal saline-alkali land, ionic balance, sodium partitioning, Potassium homeostasis

## Abstract

**Introduction:**

Agricultural development in coastal saline-alkali lands is constrained by freshwater scarcity. Utilizing brackish water for irrigation presents a viable pathway to alleviate this pressure. Honeysuckle (*Lonicera japonica* Thunb.), a salt-tolerant medicinal plant, holds promise for the ecological restoration of these areas. However, the regulatory mechanisms by which brackish water irrigation affects ionic homeostasis and physiological traits in honeysuckle remain elusive.

**Methods:**

A field experiment was conducted in the coastal saline-alkali soils of the Yellow River Delta. A randomized complete block design was employed with four brackish water irrigation regimes: T1 (rainfed control), T2 (40 mm), T3 (80 mm), and T4 (120 mm). The effects of these irrigation treatments on ion dynamics within the soil-plant system and the salt tolerance of honeysuckle were analyzed.

**Results:**

With increasing brackish water irrigation, (1) significantly reduced cation accumulation in the topsoil. Compared to T1, the soil Na^+^ content under T2, T3, and T4 decreased by 33.69%, 33.94%, and 56.53% in 2019, and by 29.53%, 41.46%, and 59.31% in 2020, respectively. Similarly, the soil K^+^ content decreased by 3.20%, 27.48%, and 38.78% in 2019, and by 33.58%, 46.77%, and 52.80% in 2020 under the same treatments. (2) In honeysuckle, selective sodium partitioning and potassium homeostasis enhanced the leaf K^+^/Na^+^ ratio. The ratio in T4 was 165.45%, 89.90%, and 48.89% higher than in T1, T2, and T3, respectively—a response driven by a 59.27% reduction in leaf Na+ and a 7.24% increase in leaf K^+^ in T4. (3) alleviated salt-induced oxidative stress in the leaves, reducing the malondialdehyde (MDA) content from 228.46 nmol•g^-1^ (T1) to 143.81 nmol•g^-1^ (T4) and decreasing hydrogen peroxide (H_2_O_2_) by 43.42%. Concurrently, the whole-plant biomass under T4 (829.56 g) exhibited an 8.8-fold increase versus T1 (94.05 g), while the total Na^+^ accumulation per plant increased from 351.66 mg (T1) to 1391.97 mg (T4).

**Discussion:**

The findings demonstrate that brackish water irrigation mitigates Na^+^ accumulation in the root zone through leaching. Honeysuckle maintains ionic homeostasis by restricting Na^+^ uptake at the root level, facilitating selective Na^+^ translocation in stems, and regulating the K^+^/Na^+^ ratio in leaves. This coordinated physiological strategy ultimately enhances biomass.

## 1 Introduction

Approximately 800 million hectares of saline-alkali soils are distributed globally across arid/semi-arid regions and coastal zones, presenting dual challenges of freshwater scarcity and secondary salinization (Montanarella et al., 2015; Baloch et al., 2023). This degradation process degrades soil structure, reduces agricultural productivity, and impedes sustainable farming practices (Kamran et al., 2021). Developing innovative scalable strategies for saline-alkali land rehabilitation has therefore become a critical priority for global food security. Current remediation approaches encompass physical interventions, chemical amendments, and biological restoration. However, these methods present persistent limitations, including secondary contamination risks and inefficient long-term efficacy (Daba, 2025). In contrast brackish water irrigation—leveraging hydrological regulation—has emerged as a promising alternative due to its “documented salt-salt-suppression” mechanism. By modulating soil ion composition, brackish water irrigation selectively reduces sodium accumulation in the root area (Shehzad et al., 2020), while maintaining potassium, simultaneously, avoiding excessive freshwater consumption and offering a sustainable pathway for coastal saline-alkali soil remediation.

Brackish water irrigation ameliorates soil salinity through multiple ionic redistribution mechanisms. Soil salts undergo transport *via* soil moisture dynamics during irrigation cycles. When irrigated with brackish water of moderate salinity, surface soil Na^+^ and Cl^-^ concentrations decline progressive with successive applications (Liu et al., 2023). Through brackish water leaching processes, surface sodium ions are mobilized and subsequently leached into deeper soil layers (Zhang and Shen, 2022), thereby decreasing salt accumulation in the root zone (Liu et al., 2016). This process not only reduces Na^+^ concentration but also optimizes soil profile ionic distribution under precision management (Yin et al., 2022). Compared with glycophytes, halophytes sequester excess Na^+^ through vacuolar compartmentalization, synthesize compatible solutes, and restrict root Na^+^ uptake (Karakas et al., 2021). Compartmentalizing Na^+^ provides a low-cost osmoticum that mitigates salt-induced water deficit (Munns and Tester, 2008), establishing salt-tolerant plants as preferred agents for saline soil restoration.

Honeysuckle (*Lonicera japonica* Thunb.) is a perennial vine highly valued for its medicinal properties, which exhibits robust environmental adaptability and considerable saline-alkali tolerance, making it highly valuable for ecological restoration. It contributes to soil structure amelioration, salt absorption, and soil-water conservation. Research showed that honeysuckle maintains intracellular ion homeostasis through selective ion uptake in roots tissues (Yan et al., 2016), and mitigates oxidative stress by enhancing the synthesis of phenolic compounds (Yan et al., 2017). The ability of honeysuckle to sustain relatively normal physiological functions under saline-alkaline stress highlights its strong capacity for homeostatic regulation and physiological adaptation. Consequently, it serves as an ideal model plant for investigating plant responses to saline-alkali soils. Previous studies have predominantly focused on brackish water irrigation for food crops such as rice and wheat, or salt-tolerant cash crops like cotton and goji berries. However, research delving into the ion response mechanisms of the medicinal plant honeysuckle (*Lonicera japonica* Thunb.) remains limited. Furthermore, the association between soil ions and plant ion homeostasis under brackish water irrigation has not yet been systematically explored. Notably, medicinal plants like honeysuckle (*Lonicera japonica* Thunb.) offer unique value for saline-alkali remediation, combing exceptional edaphic adaptability (tolerating drought and waterlogging) with high economic potential. Under salt stress, plants can enhance Na^+^ efflux through their root systems, retain K^+^ content, reduce Na^+^ accumulation in both roots and shoots, and maintain ionic homeostasis and normal physiological functions (Sun et al., 2009; Kong et al., 2012). This mitigates the adverse effects of salt stress on plant growth and development. Under salinity stress, honeysuckle stems exhibit limited Na^+^ accumulation, therefore, it cannot be used as a salt accumulator for desalination of saline-alkali soils. However, under salt stress, honeysuckle can promote root respiration, thereby facilitating calcite dissolution and increasing soil Ca^2+^ levels. This facilitates the displacement of Na^+^ ions and promotes their leaching into deeper soil layers, thus serving to desalinate the rhizosphere environment (Yan et al., 2015a; Rahman et al., 2021; Qadir et al., 2001).

Although existing research on brackish water irrigation predominantly focuses on conventional crop systems, its synergistic interactions with medicinal plants remain largely underexplored. Previous studies have primarily focused on the effects of salt stress on crop biomass, photosynthesis, and related parameters, lacking a delicate characterization of ionic dynamics across root, stem, and leaf organs in honeysuckle. This study bridges this critical knowledge gap through rigorous field experiments conducted in China's Yellow River Delta saline-alkali soils. We systematically quantify soil-root-shoot ion dynamics under graded brackish water irrigation regimes and their consequential impacts on foliar ionic composition and physiological performance in honeysuckle (*Lonicera japonica* Thunb.). Our integrated investigation elucidates honeysuckle’s desalination mechanisms to pioneer sustainable brackish water utilization strategies, concurrently alleviating freshwater scarcity in vulnerable coastal agroecosystems. Ultimately, this phytoremediation approach delivers dual agro-environmental benefits: reclaiming saline soils for enhanced agricultural productivity and fostering environmental resilience, thereby establishing synergistic ecological-economic sustainability.

## 2 Materials and methods

### 2.1 Experimental materials

**Figure 1:**
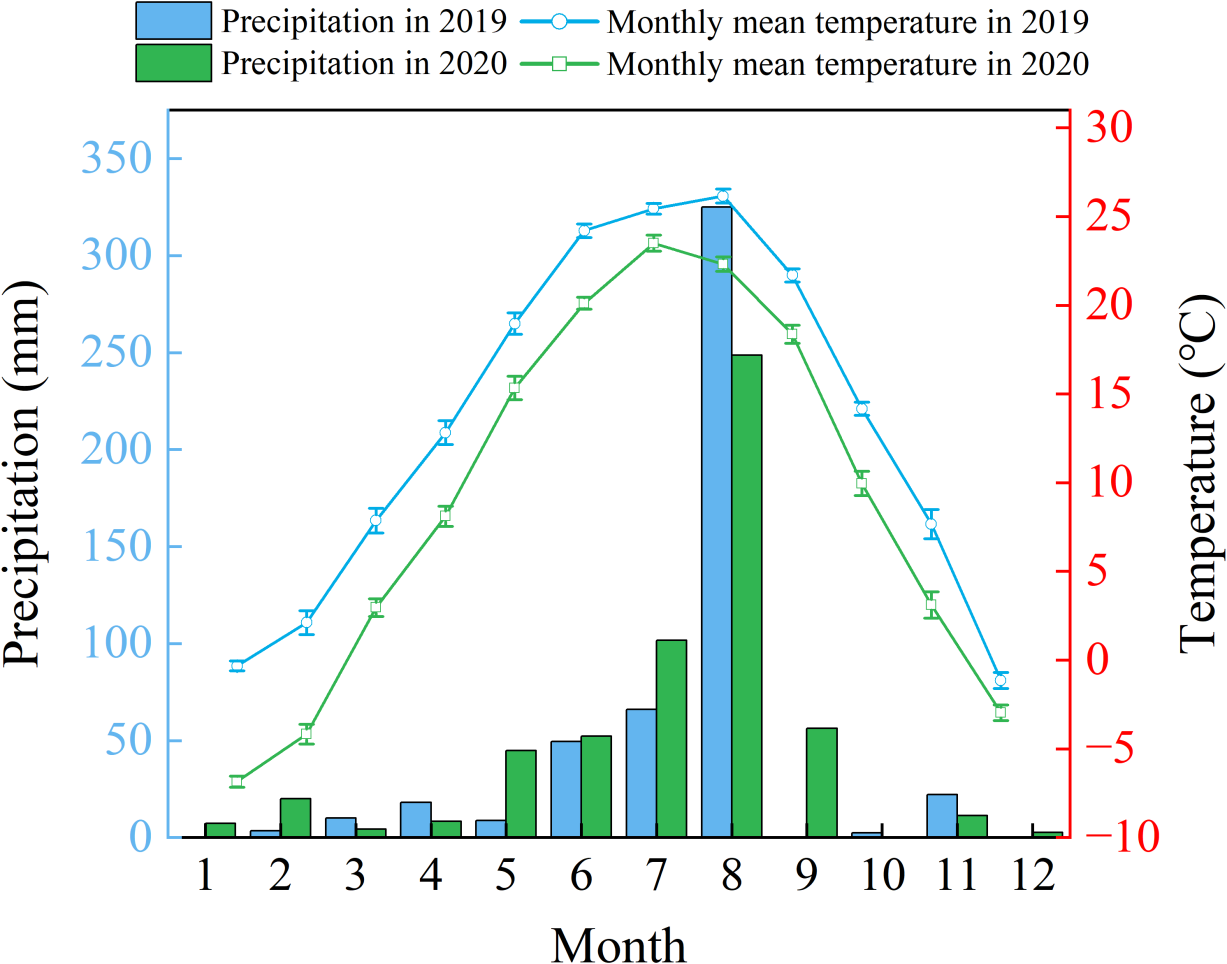
Variations in annual precipitation (2019–2020) & mean air temperature (2020) over the Yellow River Delta.

In this study, the plant material consisted of two-year-old bare-root seedlings of the tree-type honeysuckle (*Lonicera japonica* Thunb) 'Beihua No.1', supplied by Jiujianpeng Agricultural Technology Co., Ltd. in Shandong Province, China. Propagated by cuttings, this cultivar reliably expresses key agronomic traits like an extended flowering period and elevated bioactive compound content. Field trials were conducted within a coastal saline-alkali soil demonstration area at the Chinese Academy of Sciences Yellow River Delta Coastal Wetland Ecological Experimental Station (37°45'50"N, 118°59'24"E), with laboratory specimens maintained under controlled conditions. The Yellow River Delta has a warm temperate continental monsoon climate characterized by prevailing, southeasterly and northeasterly winds. Between 1961 to 2020, mean annual precipitation reached 602.98 mm (range: 357.15-1279.24 mm), while regional evaporation averaged about 1962 mm annually (He et al., 2007). Seasonally the dry period predominantly occurs from April to June, with 70% of precipitation is concentrated during July to August (**Figure 1**).

Prior to establishing brackish water irrigation plots, the soil underwent freshwater desalination pretreatment. Following rotary tillage, the experimental plots were constructed, and each plot was amended with cow dung as fertilizer (200 kg·hm^-2^). Each 3 m × 4 m rectangular plot was separated by 0.5 m buffer zones. In May 2019, uniform, well-established seedlings were transplanted at 1 m × 1 m spacing (12 plants plot). Post-transplantation management comprised application of 40 kg·hm^-2^ diammonium phosphate, freshwater irrigation, and routine phytosanitary maintenance: organic fertilization with cattle manure, manual weeding, and curative pesticide applications.

The experimental silt loam soil exhibited relatively uniform horizontal distribution. Initial characterization confirmed salinity-alkalinity parameters: electrical conductivity (EC) = 2.27 mS·cm^-1^, sodium adsorption ratio (SAR) = 7.95, and pH = 7.75. Surface soil demonstrated enrichment with Na^+^ content substantially exceeding K^+^, Ca^2+^, and Mg^2+^. The average content of Na^+^, K^+^, Ca^2+^ and Mg^2+^ in 0~80 cm soil is 1.18 mg·g^-1^, 0.04 mg·g^-1^, 0.19 mg·g^-1^, and 0.33 mg·g^-1^, respectively. Irrigation water quality monitoring revealed brackish characteristics: electrical conductivity (EC) values ranged between 4.17-5.19 mS·cm^-1^ (2019) and 4.77-5.09 mS·cm^-1^ (2020), while salinity levels fluctuated from 2.31‰-2.48‰ and 2.7‰-2.88‰ in respective years. The water was alkaline, with pH spanning 8.15-8.89 in 2019, whereas 2020 measurements showed a narrower range of 8.73-8.89. The brackish water exhibited the following characteristics: total nitrogen (TN) 0.83-3.42 mg·L^-1^, ammonium nitrogen (NH_4_^+^-N) 0.3-2.4 mg·L^-1^, total phosphorus (TP) 0.03-0.28 mg·L^-1^. Major ion concentrations were sodium (Na^+^) 1050 mg·L^-1^ on average (range: 608.9-1906.03 mg·L^-1^), potassium (K^+^) 14.07 mg·L^-1^ on average (range: 12.54-15.30 mg·L^-1^), calcium (Ca^2+^) 69.99 mg·L^-1^ on average (range: 59.8-86.44 mg·L^-1^), and magnesium (Mg^2+^) 2.68 mg·L^-1^ on average (range: 2.10-3.08 mg·L^-1^).

### 2.2 Experimental design

The brackish water irrigation experiment commenced in late June 2019 with four treatments: T1 (control, rainfed without irrigation), T2 (40 mm), T3 (80 mm), and T4 (120 mm). Employing a randomized complete block design, each treatment had four replicates. Uniform irrigation schedules and field management practices, including weeding and insecticide application were maintained across treatment. Irrigation events occurred on 25 June, 29 July, and 27 September 2019, followed by 29 April and 20 June 2020.

### 2.3 Soil sampling and physicochemical property determination

Sampling occurred five days after each irrigation event during irrigated months, and during the middle or late part of the month during non-irrigation months. Composite sample were formed three S-pattern, points per plot. Soil cores were stratified into 0–20 cm, 20–40 cm, 40–60 cm, and 60–80 cm depths. Air-dried, sieved, soils underwent aqueous extraction 5:1 water-to-soil ratio. Homogenized suspensions were vortexed (30 min), diluted 20-fold and filtered 0.45 μm. The quantification of Na^+^, K^+^, Mg^2+^, and Ca^2+^ was performed using ion chromatography (Michalski, 2006).

### 2.4 Monitoring of growth parameters in honeysuckle (*Lonicera japonica* Thunb.)

#### 2.4.1 Biomass

Plant sampling occurred in October 2020 at the end of the growing season. Two representative plants from each treatment group were selected and excavated. Leaf, stems, and roots fresh weights were recorded upon excavation before oven-drying at 60°C to constant weight for dry biomass determination (Dabo et al., 1987). Root system analysis employed a 30 × 30 cm quadrat positioned around root crown. Soil-root samples were collected at 10 cm intervals from 0–40 cm depth. All roots were thoroughly washed, oven-dried at 60°C to constant mass, and weighed.

#### 2.4.2 Base diameter, plant height and leaf area index

Stem basal diameter was measured at 5 cm above the soil surface using a vernier caliper. Plant height was determined as the distance from the base to the apical growing point using a measuring tape. The leaf area index (LAI) of individual plants was measured with an LP-80 LAI meter. These measurements were taken monthly in mid- to late-month periods, aligning with the soil sampling schedule. For each quadrat, all plants were measured for basal diameter, plant height, and LAI, and the mean values were used as representative quadrat-level data.

#### 2.4.3 Determination of Na^+^ and K^+^ content

Na^+^ and K^+^ contents in roots, stems, and leaves of October 2020 honeysuckle samples were quantified. Dried plant tissue (0.1 g) was powdered, transferred to sealed tubes, and boiled in 25 ml deionized water at 100°C for 2 hours. Aqueous extracts were analyzed by flame atomic absorption spectrophotometry (Song et al., 2011).

#### 2.4.4 Determination of malondialdehyde and hydrogen peroxide H_2_O_2_ contents

Fresh leaf sample (0.2 g) was flash-frozen in liquid nitrogen and mechanically homogenized in 4 mL 0.1% trichloroacetic acid (TCA). After centrifugation (12,000 × g for 10 min at 4°C) the supernatant was collected for subsequent analyses. Lipid peroxidation was assessed by quantifying malondialdehyde (MDA) content through the thiobarbituric acid (TBA) reaction (Heath and Packer, 1968). For hydrogen peroxide (H_2_O_2_) determination, 1 ml supernatant was reacted with 1 ml 0.1 mM potassium phosphate buffer (pH 7.0) and 2 ml of 1 mM potassium iodide (KI), with absorbance measurement at 390 nm (Alexieva et al., 2001).

### 2.5 Data processing and analysis

#### 2.5.1 Translocation factor of honeysuckle

(1) $$\begin{array}{c} {\text{Na}}^{+}\;\text{accumulation}\\ =(\text{leaf dry weight}\times {\text{leaf Na}}^{+}\;\text{concentration})\\ +(\text{stem dry weight}\times {\text{stem Na}}^{+}\;\text{concentration})\\ +(\text{root dry weight}\times {\text{root Na}}^{+}\;\text{concentration}) \end{array}$$

(2) $$\text{TF}1={\text{stem Na}}^{+}\text{concentration}/{\text{root Na}}^{+}\text{ concentration}$$

(3) $$\text{TF}2={\text{leaf Na}}^{+}\text{concentration}/{\text{root Na}}^{+}\text{ concentration}$$

The unit of Na_+_ accumulation is mg•plant_-1_ (Equation 1). TF1 represents the Na+ translocation factor from roots to stem in honeysuckle (Equation 2), TF2 represents the Na_+_ translocation factor from roots to leaves (Equation 3) (Yan et al., 2015b).

#### 2.5.2 Statistical analysis

Systematic statistical analyses evaluated brackish water irrigation effects on soil-plant responses. Three core aspects were assessed *via* one-way ANOVA and Duncan multiple comparison method: (1) spatial cation redistribution in soil matrices, (2) Na^+^ partitioning across honeysuckle root-stem-tissues, and (3) biomass allocation dynamics coupled with oxidative stress biomarkers (MDA and H_2_O_2_ concentrations). Statistical analyses used SPSS (*α* = 0.05, 95% confidence interval). A multi-way ANOVA was employed to examine the effects of rainfall, treatment, year, and their interactions on the concentrations of Na^+^, K^+^, Mg^2+^, and Ca^2+^ at four soil depths (20 cm, 40 cm, 60 cm, and 80 cm). The data for K^+^, Mg^2+^, and Ca^2+^ at 20 cm, Ca^2+^ at 40 cm, K^+^ and Mg^2+^ at 60 cm, and K^+^ and Mg^2+^ at 80 cm were subjected to logarithmic transformation. The NMDS analysis incorporated key parameters including root Na^+^ content, stem Na^+^ content, leaf Na^+^ content, root biomass, stem biomass, leaf biomass, total biomass, MDA, and H_2_O_2_. The metaMDS yielded a stress value of 0.009, PERMANOVA confirmed significant treatment effects (*F* = 123.328, *P* = 0.001). Figures were generated using in Origin and Bioinformatic Cloud.

## 3 Results

### 3.1 Brackish water irrigation effects on soil cation dynamics

**Figure 2:**
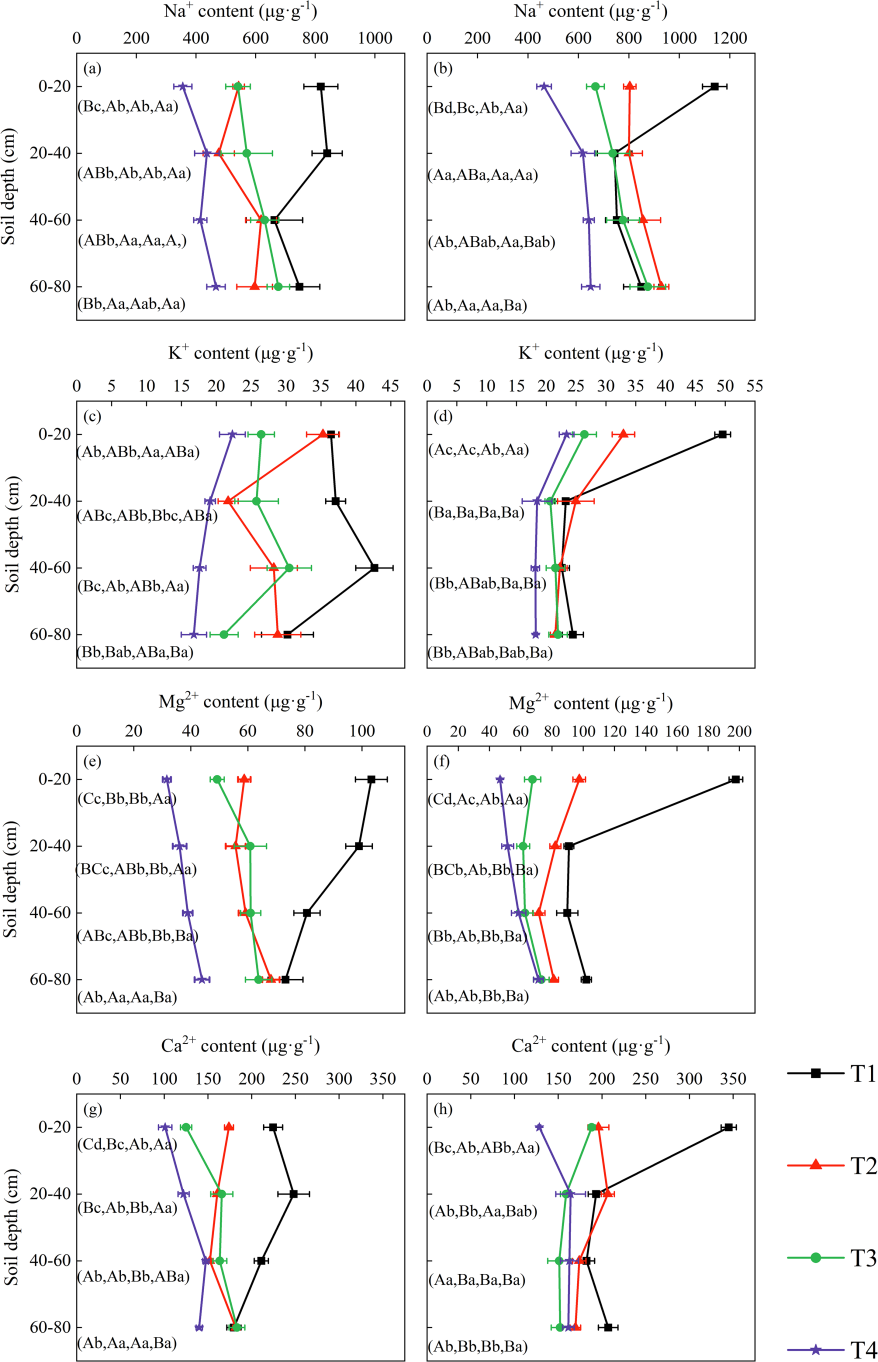
Soil cation distribution in the profile under brackish water irrigation treatments for 2019 **(a, c, e, g)** and 2020 **(b, d, f, h)** (Mean ± SE, n = 4). T1 (control, no irrigation); T2 (40 mm); T3 (80 mm); T4 (120 mm). Uppercase letters indicate significant differences (*P* < 0.05) between soil layers within the same treatment; lowercase letters denote significant differences within the same soil layers across treatments.

In the topsoil layer, the contents of the four cations showed a significant decreasing trend with increasing volumes of brackish water irrigation in both 2019 and 2020 (**Figures 2**). Treatment, year, and their interaction all exerted highly significant effects on surface soil ion concentrations (**Supplementary Table S1**). Compared to T1, the Na^+^ content in T2, T3, and T4 decreased by 33.69%, 33.94%, and 56.53% in 2019, and by 29.53%, 41.46% and 59.31% in 2020, respectively. The K^+^ content under T2, T3, and T4 treatments decreased by 3.20%, 27.48%, and 38.78% in 2019, and by 33.58%, 46.77% and 52.80% in 2020, respectively, compared to T1. The contents of Mg^2+^ and Ca^2+^ also decreased over the two years as irrigation volume increased, with significant differences observed among the various treatments.

As soil depth increased, the contents of all four cations exhibited a decreasing trend under the non-irrigation treatment. Under irrigation treatments, Na^+^ content increased to varying degrees with depth in both 2019 and 2020, whereas K^+^ content decreased across all treatments. Mg^2+^ content showed a decreasing trend in the T2 treatment in 2020 but increased in the other treatments with increasing soil depth. The variation in Ca^2+^ content was more dynamic: overall, it displayed an increasing trend across treatments in 2019, while in 2020, it increased under the T4 treatment but decreased under T2 and T3 treatments. With increasing soil depth, the influences of rainfall and year progressively decreased, and their interactions became more complex or non-significant. In contrast, the treatment effect on soil ions remained highly significant (**Supplementary Table S1**).

**Figure 3:**
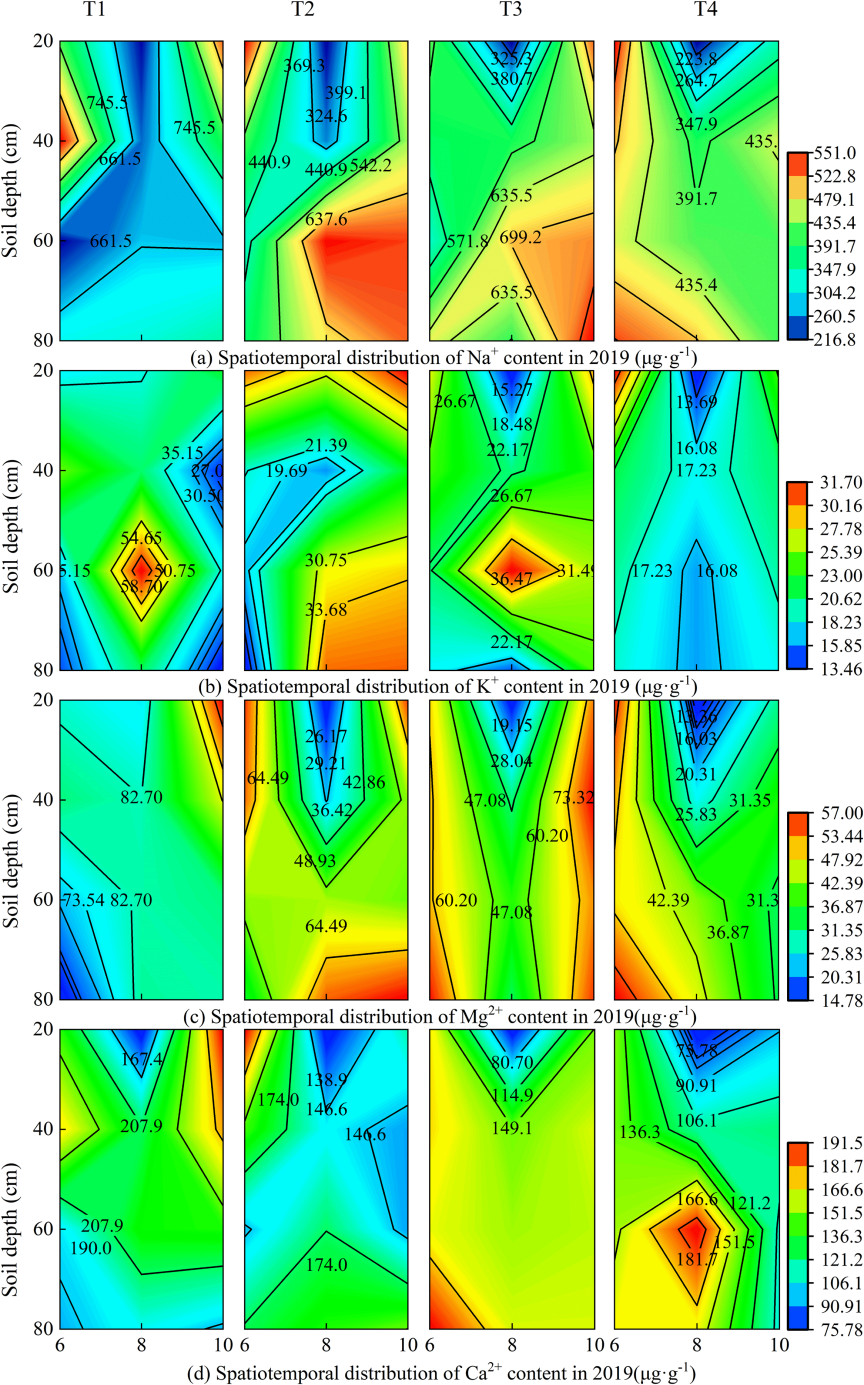
Spatiotemporal distribution of soil cations under different brackish water irrigation treatments in 2019 (Mean ± SE, n = 4). T1 (control, no irrigation); T2 (40 mm); T3 (80 mm); T4 (120 mm). Subpanels **(a)**, **(b)**, **(c)**, and **(d)** represent the contents of Na^+^, K^+^, Mg^2+^, and Ca^2+^ in different months of 2019, respectively.

**Figure 4:**
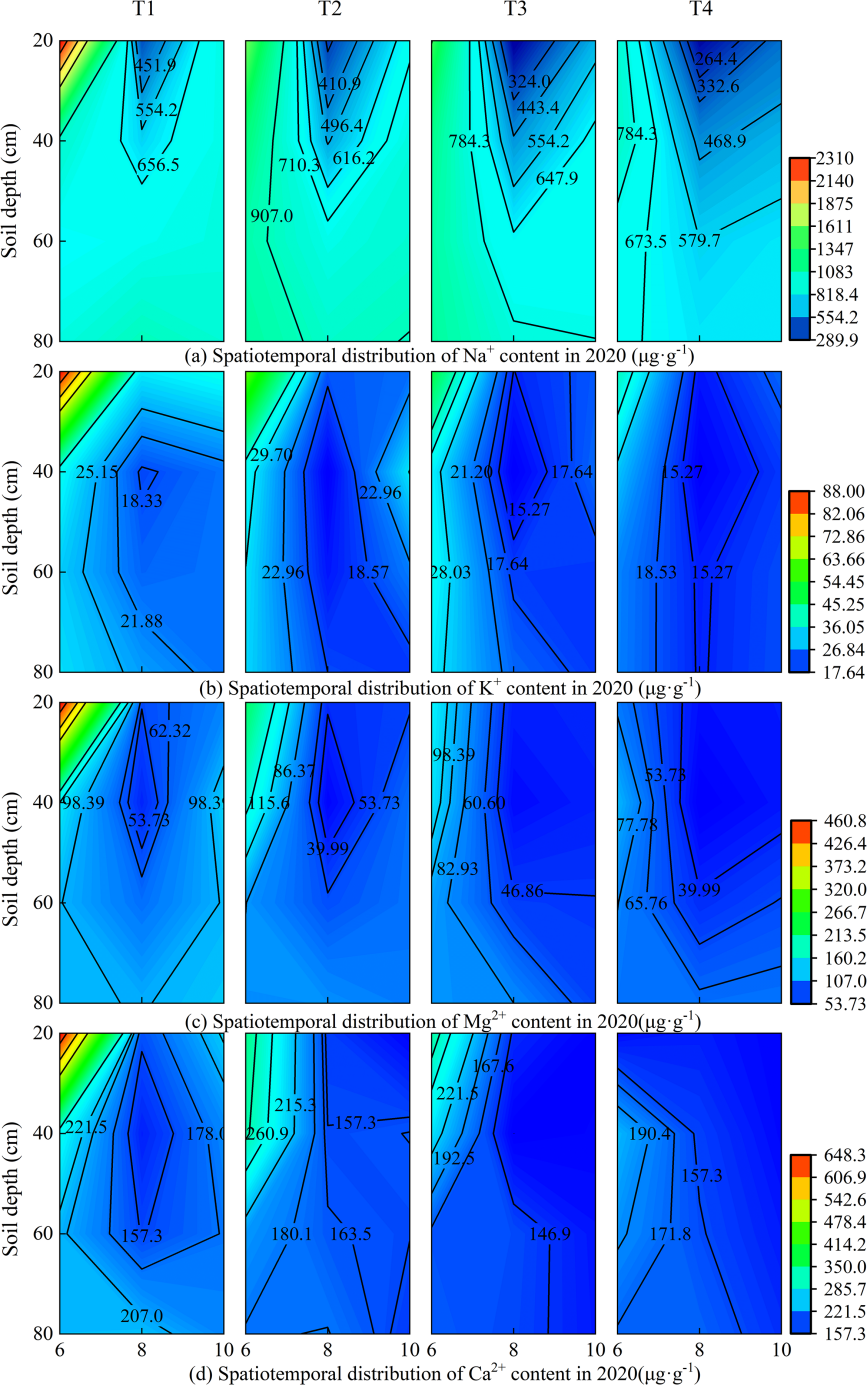
Spatiotemporal distribution of soil cations under different brackish water irrigation treatments in 2020 (Mean ± SE, n = 4). T1 (control, no irrigation); T2 (40 mm); T3 (80 mm); T4 (120 mm). Subpanels (a), (b), (c), and (d) represent the contents of Na^+^, K^+^, Mg^2+^, and Ca^2+^ in different months of 2020, respectively.

Under different brackish water irrigation volumes, the four cations exhibited distinct seasonal and spatial variations in both 2019 and 2020 (**Figures 3**, **4**). Rainfall, treatment, and year each independently exerted a highly significant influence on the cations in the surface soil layer (**Supplementary Table S1**). In the months of June, August, and October, the cation contents decreased with increasing irrigation volume across all soil layers. In both years, the cation contents in the topsoil were relatively high in June. Compared to June, the contents of all cations decreased significantly in August of both 2019 and 2020. By October, the cation concentrations in the topsoil had increased relative to August. Rainfall, year, and their interactions exerted highly significant effects on the seasonal variations in ion concentrations (**Supplementary Table S1**). Under the non-irrigation treatment, the ion content in October 2019 was higher than that in June, whereas in 2020, it was lower than in June. Under irrigation treatments, in October 2019, the Na^+^ content under the T3 treatment was higher than in June, and the K^+^ content under T2 and T3 treatments in October was also higher than in June, while the other treatments remained lower than the June levels. In contrast, in 2020, the topsoil ion contents in October were generally lower than those in June.

### 3.2 Brackish water irrigation effects on ion accumulation in honeysuckle

**Figure 5:**
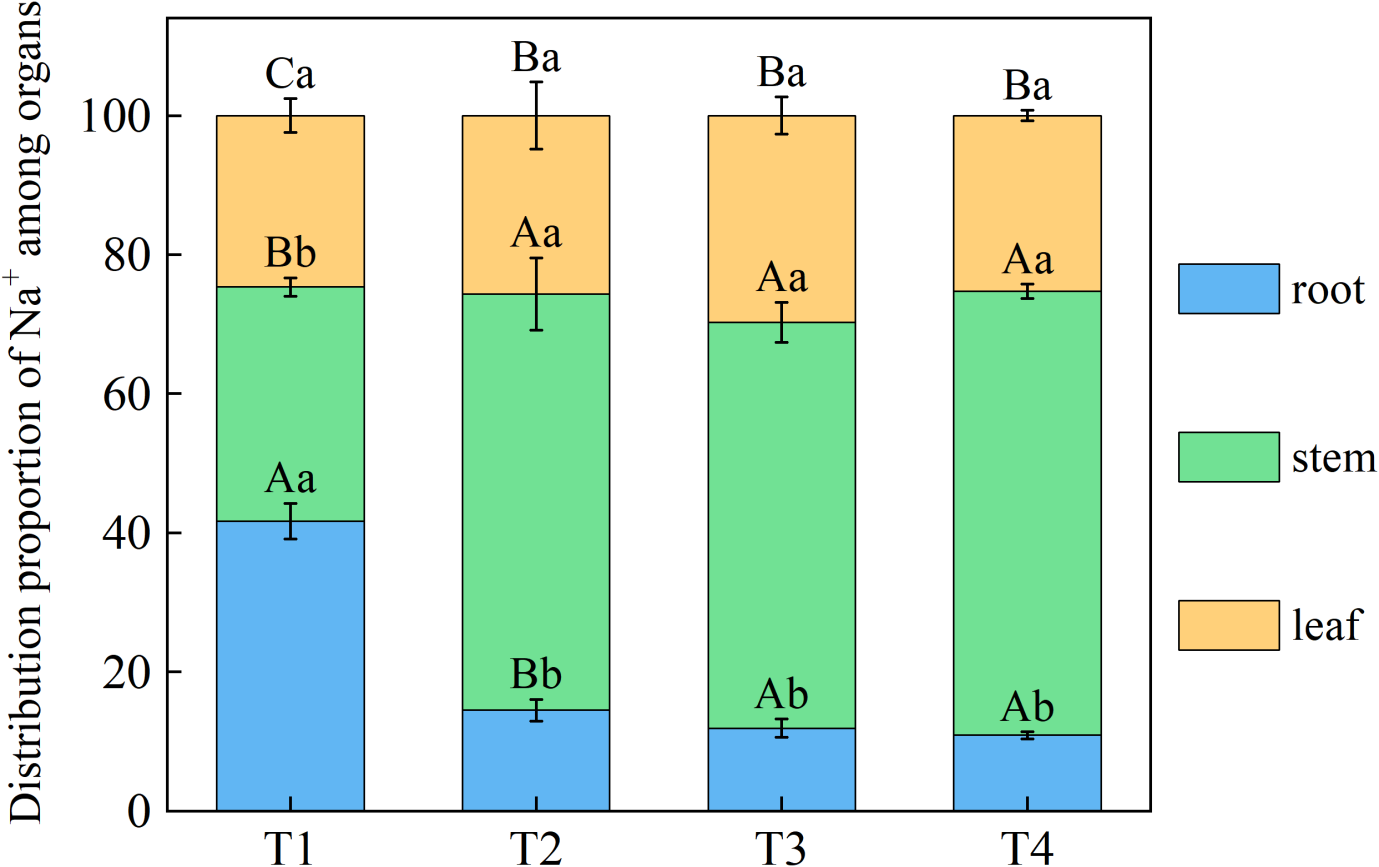
Effects of different brackish water irrigation levels on Na^+^ distribution in honeysuckle organs (Mean ± SE, n = 4). T1 (control, no irrigation); T2 (40 mm); T3 (80 mm); T4 (120 mm). Uppercase letters indicate significant differences among different organs under the same treatment; lowercase letters denote significant differences among different treatments for the same organ (*P*< 0.05).

**Figure 6:**
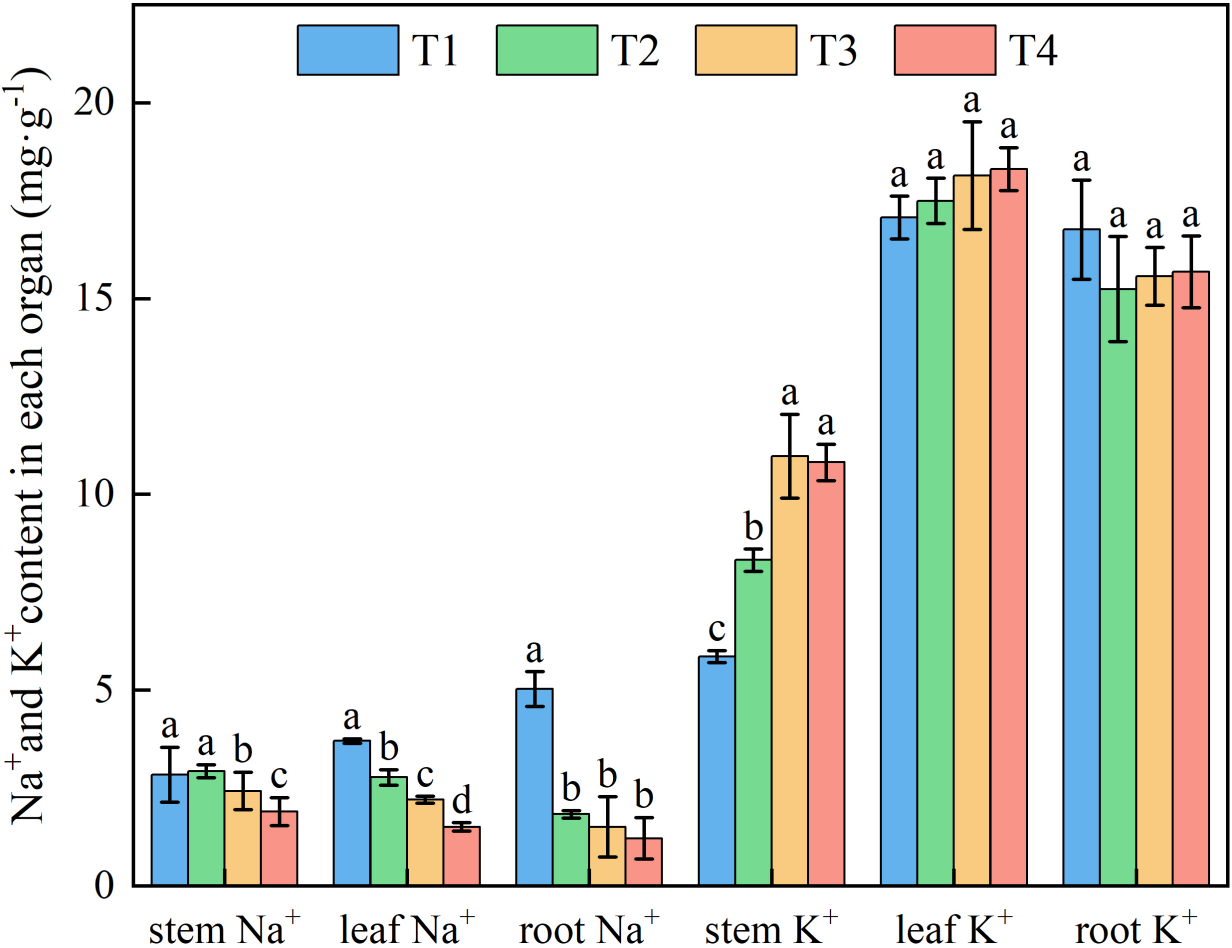
Effects of different brackish water irrigation amounts on Na^+^ and K^+^ contents in honeysuckle (Mean ± SE, n = 4). T1 (control, no irrigation); T2 (40 mm); T3 (80 mm); T4 (120 mm). Different letters indicate significant differences between treatments (*P* < 0.05).

Brackish water irrigation significantly altered Na^+^ distribution patterns in honeysuckle tissues, with root-to-leaf gradients showing progressive depletion as irrigation volume increased (**Figure 5**). Root Na^+^ decreased incrementally across treatments, T1 accumulated 63.61%, 70.05%, and 75.80% higher than T2, T3, and T4, respectively. Significant differences in Na^+^ were observed between the T1 stem and T2, T3, T4 (*P* < 0.05). Compared to T1, leaf Na^+^ in T2, T3, and T4 decreased by 25.09%, 40.60%, and 59.27%, respectively (**Figure 6**).

**Figure 7:**
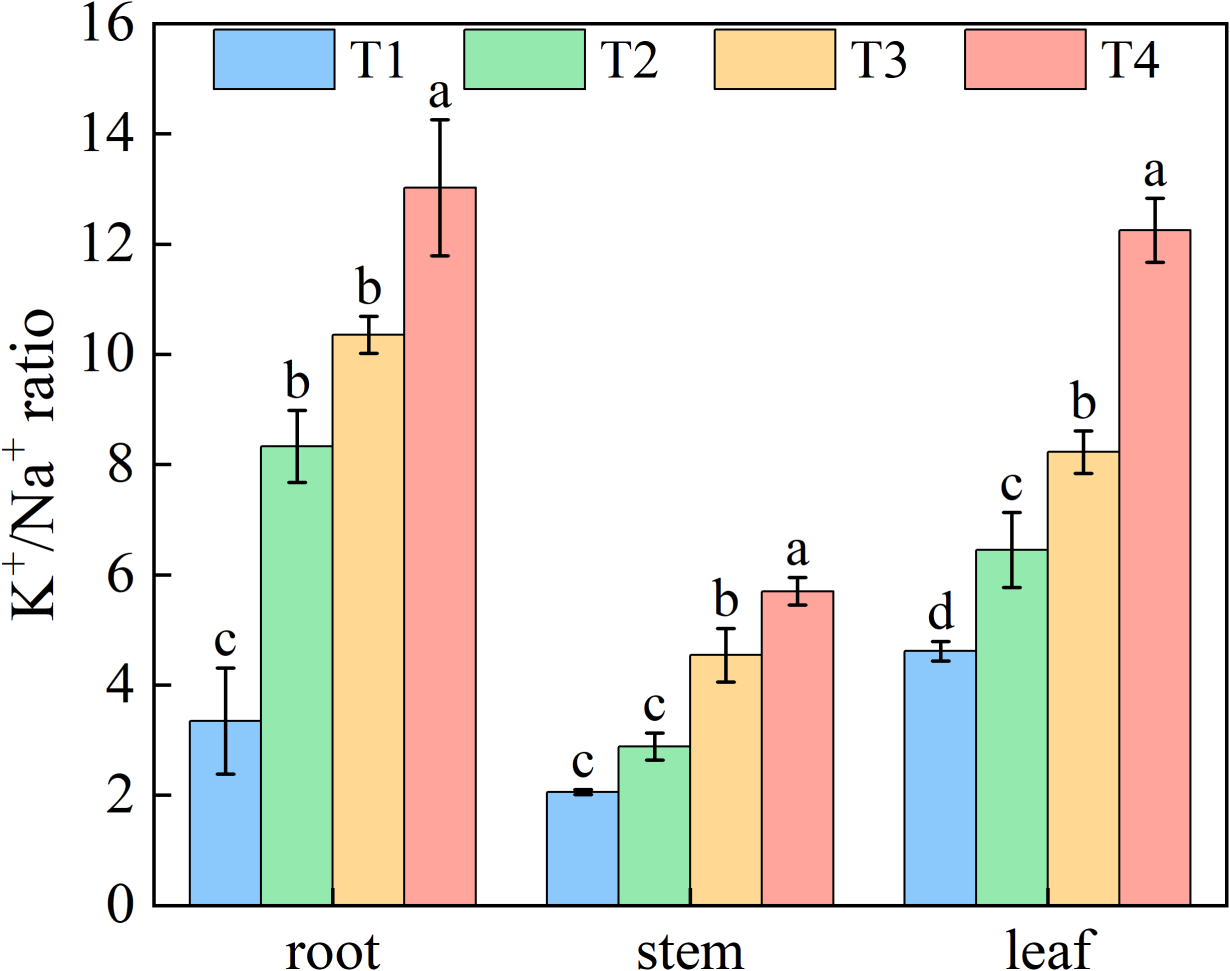
Effects of brackish water irrigation amounts on Potassium-Sodium Balance in honeysuckle plants (Mean ± SE, n = 4). T1 (control, no irrigation); T2 (40 mm); T3 (80 mm); T4 (120 mm). Different letters indicate significant differences between treatments (*P*< 0.05).

Increasing brackish water irrigation progressively reduced Na^+^ content in honeysuckle. Conversely, while root K^+^ declined with irrigation intensity, stem and leaf K^+^ levels exhibited progressive increases. Under each treatment, stem K^+^ remained persistently lower than roots and leaves (**Figure 6**). Elevated irrigation amounts proportionally increased tssues K^+^/Na^+^ ratio (**Figure 7**). Root tissues showed the high K^+^/Na^+^ ratio under T4 (12.88). Leaf displayed significant treatment differences, with K^+^/Na^+^ ratio escalating with irrigation levels. Specifically, T4 exhibited 165.45%, 89.90%, and 48.89% higher ratio than T1, T2, and T3, respectively.

### 3.3 Effects of brackish water irrigation on MDA and H_2_O_2_ in honeysuckle

**Figure 8:**
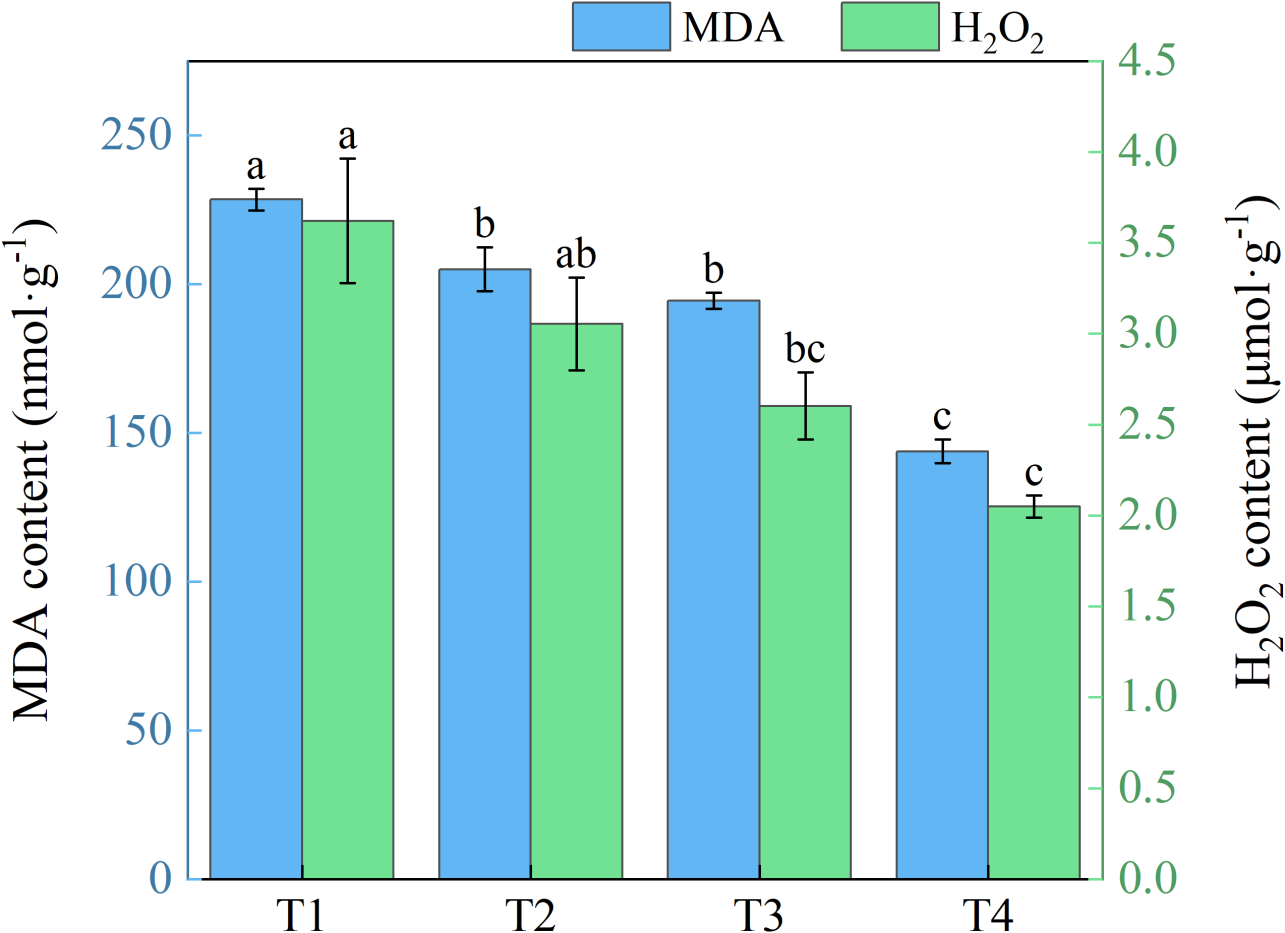
Effects of different brackish water irrigation levels on MDA and H_2_O_2_ contents in honeysuckle plants (Mean ± SE, n = 4). T1 (control, no irrigation); T2 (40 mm); T3 (80 mm); T4 (120 mm). Different letters indicate significant differences between treatments (*P* < 0.05).

With expanding use of brackish water irrigation, honeysuckle leaves exhibited a gradual decrease in both MDA and H_2_O_2_ contents (**Figure 8**). T1 showed 10.28%, 14.90%, and 37.05% higher MDA than T2, T3, and T4, with marked reduction across treatments. T1 showed 15.69%, 28.14%, and 43.42% higher H_2_O_2_ than T2, T3, and T4, respectively. Significant differences were observed between T1 and T3, T4, but not between T1 and T2.

### 3.4 Brackish water irrigation effects on biomass and Na^+^ translocation in honeysuckle

As the irrigation of brackish water irrigation increased, the basal diameter, plant height, and leaf area index of honeysuckle showed significant increases (**Supplementary Figure S1**), significant differences were observed between treatment T4 and treatments T3, T2, T1. Biomass increased dependently with brackish water irrigation intensity (**Table 1**). T4 achieved 829.560 g total biomass 8.82, 5.09, and 1.97-fold higher than T1, T2, and T3. Root, stem, and leaf biomass peaked under T4 treatments. Root biomass in these treatments exceeded T1 by 33.62% (T2), 180.74% (T3) and 325.31% (T4). Stem biomass in T2, T3, and T4 increased by 138.22%, 515.88%, and 1028.54%, respectively, compared with T1. T2, T3, and T4 leaf biomass increased by 140.69%, 507.02%, and 918.94%, respectively, compared to T1. T4 was significantly higher than the other three groups, followed by T3 which showed significantly higher values than T1 and T2 but remained significantly lower than T4. No significant difference was observed between T1 and T2, both of which were significantly lower than T3 and T4.

**Table 1:** Effects of different brackish water irrigation levels on biomass and translocation factor in honeysuckle (mean ± SE, n = 4).

Indicators	T1	T2	T3	T4
Base diameter (mm)	15.505 ±0.18 c	18.022 ±0.31 b	18.755 ±0.29 b	21.376 ±0.44 a
Plant height (cm)	42.786 ±1.25 c	49.331 ±1.67 b	53.618 ±1.72 b	61.402 ±1.06 a
Leaf area index	0.776 ±0.04 c	1.103 ±0.07 c	1.618 ±0.12 b	2.565 ±0.16 a
Root biomass (g)	29.358 ±2.16 Cc	39.228 ±5.7 Cc	82.42 ±7.20 Cb	124.863 ±6. 96 Da
Stem biomass (g)	41.573 ±3.08 Bc	99.034 ±5.06 Bc	256.041 ±31.88 Bb	469.169 ±21.98 Ba
Leaf biomass (g)	23.115 ±1.62 Cc	47.313 ±11.41 Cc	140.105 ±2.67 Cb	235.528 ±12.21 Ca
Total biomass (g)	94.045 ±5.40 Ad	185.574 ±18.63 Ac	478.565 ±35.53 Ab	829.560 ±32.55 Aa
Na^+^ accumulation (mg·plant^-1^)	351.166 ±20.85 c	489.520 ±34.31 c	1051.129 ±69.78 b	1391.971 ±41.19 a
TF1	0.58 ±0.05 b	1.60 ±0.05 a	1.62 ±0.05 a	1.57 ±0.09 a
TF2	0.75 ±0.06 b	1.53 ±0.15 a	1.47 ±0.11 a	1.25 ±0.13 a

Uppercase letters indicate significant differences among different organs under the same treatment, while lowercase letters denote significant differences among different treatments within the same organ (*P*< 0.05) (Mean ± SE, n = 4). These measurements were conducted during the final sampling at the end of the growing season.

The overall Na^+^ accumulation in honeysuckle increased significantly with rising irrigation volume (**Table 1**). Compared to T1, T2, T3, and T4 showed increases of 39.40%, 199.33%, and 296.39%, respectively. T4 exhibited the highest accumulation, followed by T3. No significant difference was observed between T1 and T2, and both were significantly lower than T3 and T4. Significant differences were observed in the Na^+^ transporter factor (TF) of honeysuckle between non-irrigated and irrigated treatments (**Table 1**). Root-to-stem Na^+^ translocation factor (TF1) was significantly depressed in T1 versus other treatments. T2, T3, and T4 showed 175.9%, 179.3%, and 170.7% increases respectively compared to T1 (*P* < 0.05). The root-to-leaf translocation factor (TF2) increased by 104%, 96%, and 66.7% in T2, T3, and T4, respectively, compared to T1. Under irrigation treatments, TF1 and TF2 tended to decrease with increasing irrigation amounts, although the differences were not significant.

### 3.5 NMDS and correlation analysis of brackish water irrigation

**Figure 9:**
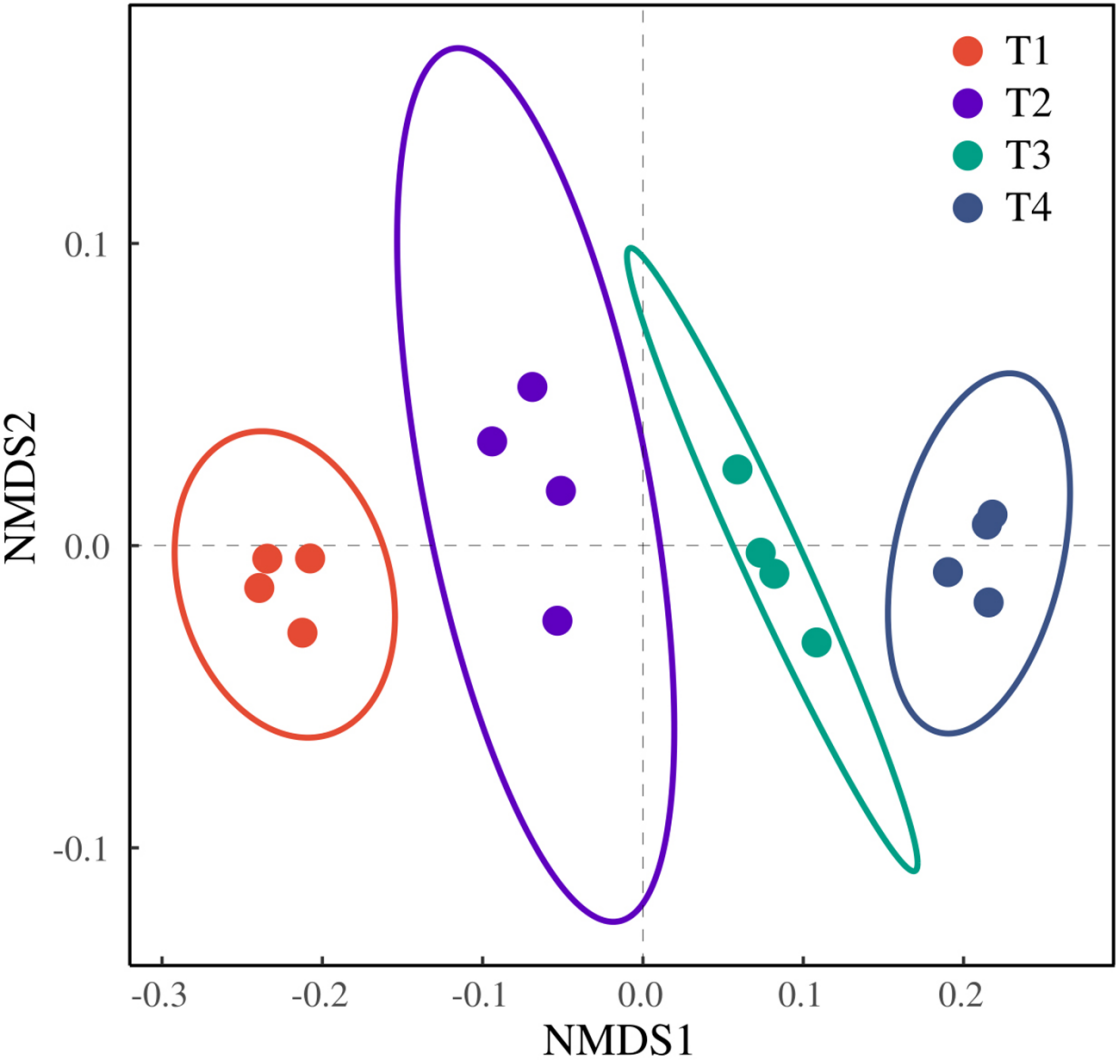
NMDS analysis of honeysuckle indicators under different brackish water irrigation treatments (Mean ± SE, n = 4).

The ordination revealed a clear separation among the four treatments without overlap, indicating distinct differences. T1 and T4 formed tight clusters along the NMDS2 axis, suggesting high within-treatment homogeneity. In contrast, T2 and T3 exhibited a more dispersed distribution, reflecting a certain degree of heterogeneity among their replicates. Notably, T1 and T4 were positioned farthest apart on NMDS1, demonstrating the most pronounced difference. The closer proximity of T2 and T3 on the ordination plot indicates a smaller difference between them (**Figure 9**).

**Figure 10:**
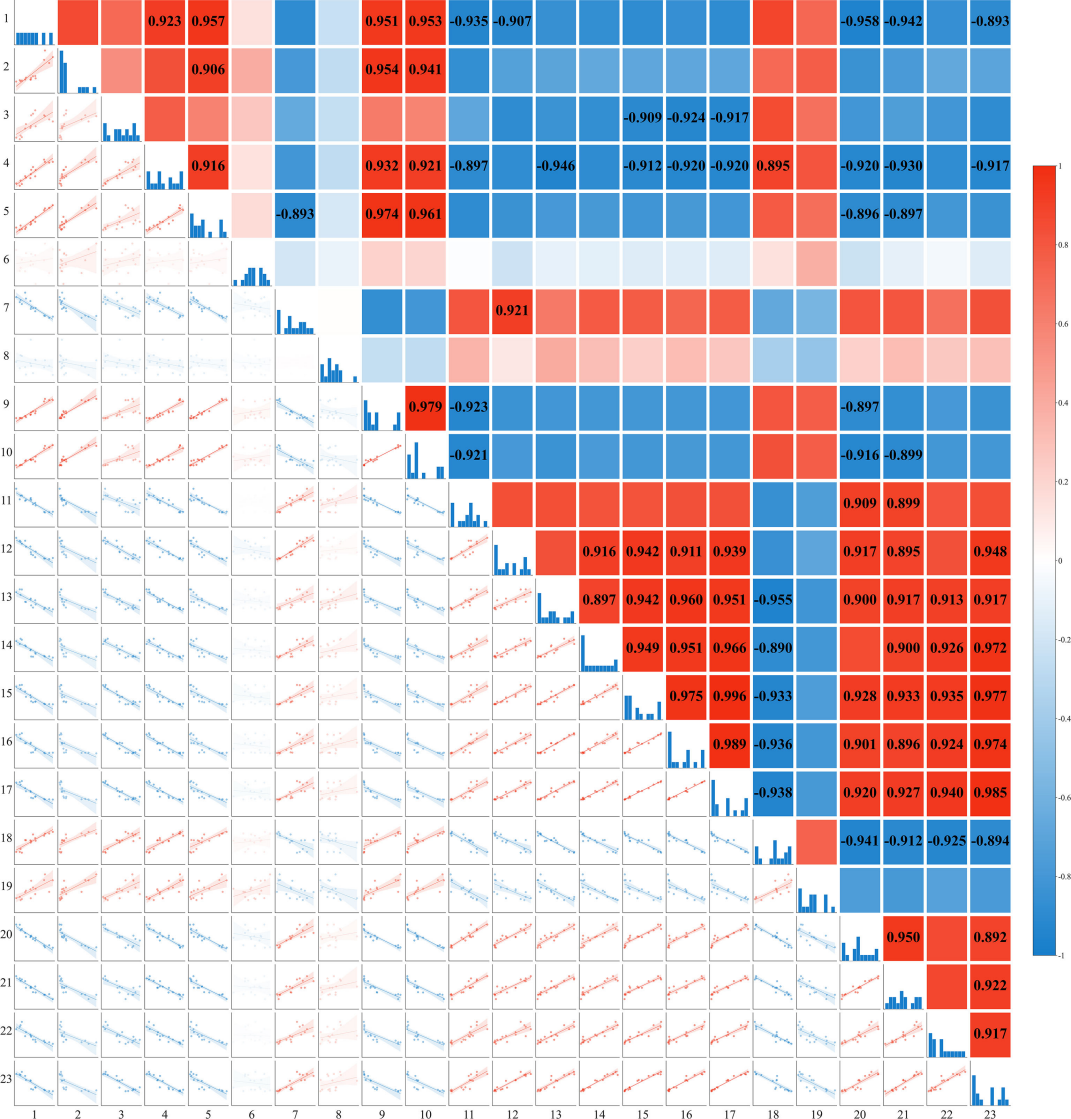
Correlation analysis of soil ion content with growth and physiological indices of honeysuckle under different irrigation levels (Mean ± SE, n = 4). 1: 0-20 cm Na^+^ content; 2-4: Na^+^ content in roots, stems, and leaves; 5: 0-20 cm K^+^ content; 6-8: K^+^ content in roots, stems, and leaves; 9: 0-20 cm Mg^2+^ content; 10: 0-20 cm Ca^2+^ content; 11-13: K^+^/Na^+^ ratio in roots, stems, and leaves; 14-17: root, stem, leaf, and total biomass; 18: MDA content; 19: H_2_O_2_ content; 20: base diameter; 21: plant height; 22: leaf area index; 23: Na^+^ accumulation.

**Figure 10** depicts the correlations among surface soil cations (Na^+^, K^+^, Mg^2+^, Ca^2+^), the concentrations of Na^+^ and K^+^ in various honeysuckle organs, organ biomass and total biomass, MDA and H_2_O_2_ content, leaf area index, basal diameter, plant height, and Na^+^ accumulation. The Na^+^ content in the surface soil showed a significant positive correlation with the contents of K^+^, Mg^2+^, and Ca^2+^ (*P* < 0.01), indicating ionic co-variation. The K^+^/Na^+^ ratio in the organs was positively correlated with biomass (*P* < 0.01). Biomass was significantly positively correlated with Na^+^ accumulation (*P* < 0.01). MDA content exhibited significant positive correlations with surface soil Na^+^, K^+^, Mg^2+^, and Ca^2+^ (*P* < 0.01). The leaf K^+^/Na^+^ ratio showed significant positive correlations with both the leaf area index and Na^+^ accumulation. The K^+^ content in the stems showed a highly significant negative correlation (*P* < 0.01) with the Na^+^ content in all honeysuckle organs. Furthermore, the K^+^/Na^+^ ratio across all organs was negatively correlated with both the surface soil cation (Na^+^, K^+^, Mg^2+^, Ca^2+^) content (*P* < 0.01) and MDA (*P* < 0.01).

## 4 Discussion

### 4.1 Effects of brackish water irrigation on ionic homeostasis in soil and honeysuckle plants

The variations in soil ion content observed in 2019 and 2020 demonstrate that brackish water irrigation significantly reduced ion levels in the surface soil, achieving effective leaching of salts in both years (**Figure 2**). Substantial seasonal variations occurred, characterized by salt accumulation in spring, desalination in summer, and slight salt accumulation in autumn. This dynamic process was strongly governed by rainfall, year, and their interaction. Existing studies indicate that increasing brackish water irrigation volume facilitates downward migration of peak salt accumulation within the soil profile (Zhang and Shen, 2022; Wei et al., 2019; Lou et al., 2025). Irrigation with brackish water increased moisture in soil macropores, promoting soil solution flow and leaching topsoil cations downward (Wang et al., 2019; Gonçalves et al., 2010). Soil Na^+^ exhibits relatively weak adsorption forces on soil particle surfaces, rendering it highly leachable and prone to downward migration (Chen et al., 2022a; Libutti et al., 2019). In surface soil layer, Na^+^ content showed significant positive correlations with K^+^, Ca^2+^, and Mg^2+^ (**Figure 10**). Brackish water typically contains Ca^2+^ and Mg^2+^, which compete with Na^+^ for adsorption sites on soil colloid (FAO and AWC, 2023). Due to their higher valence, Ca^2+^ and Mg^2+^ exhibit stronger colloidal adsorption capacity (Zhang and Norton, 2002), gradually displacing adsorbed Na^+^ and K^+^ ions into soil solution. Subsequent irrigation leaching facilitates Na^+^ and K^+^ removal, promoting their release (Abdelghany et al., 2022; Xue et al., 2022). Meanwhile, Mg^2+^ and Ca^2+^ undergo continuous adsorption-desorption equilibrium on colloidal surfaces. This complex interplay can induce simultaneous variations in multiple cations, manifesting as positive correlation (Li et al., 2019; Jin et al., 2022). The treatment and its interaction with year exerted significant effects on the concentrations of multiple ions, providing statistical support for the impact of annual irrigation practices on the adsorption-displacement-leaching mechanism, thereby collectively modulating the coordinated variations of multiple cations.

Soil ionic changes directly influenced plant ion uptake. Brackish water irrigation significantly reduced Na^+^ accumulation in honeysuckle roots stem, and leaves (**Figure 6**). While previous studies reported increased tissue Na^+^ with higher brackish water salinity or irrigation input (Sedaghathoor and Abbasnia Zare, 2019; Jin et al., 2022; Munns and Tester, 2008; Abd El Baki et al., 2025), our study revealed that Na^+^ in the organs of honeysuckle decreased with increasing irrigation volume. Given consistent irrigation water mineralization, increased water input raised topsoil moisture, diluting Na^+^ concentration. Concurrent downward leaching reduced topsoil Na^+^ content, thus decreasing plant uptake (Berrueta et al., 2023; Valdez-Aguilar et al., 2009). In honeysuckle, root K^+^ decreased with irrigation volume, whereas stem K^+^ increased and leaf K^+^ remained stable (**Figure 6**). The K^+^/Na^+^ ratio increased progressively, particularly in roots (**Figure 7**). Elevated irrigation volume enhanced Na^+^ influx into root cells, neutralizing negative charges and triggering membrane depolarization that induced K^+^ efflux (Sedaghathoor and Abbasnia Zare, 2019). Na^+^ content across different organs of honeysuckle showed a significant negative correlation with K^+^ content in the stems (**Figure 10**). Competition for identical binding sites enables Na^+^ exclusion from xylem *via* HKT transporters, reducing stem Na^+^ accumulation while facilitating preferential K^+^ uptake (Munns and Tester, 2008; Horie et al., 2009; Fu et al., 2025).

The key to plant salt tolerance lies in restricting Na^+^ influx, selectively absorbing K^+^, maintaining optimal K^+^/Na^+^ ratio, and thereby sustaining growth (Qu and Han, 2022; Pramila et al., 2019). The K^+^/Na^+^ ratio in all organs was significantly negatively correlated (*P* < 0.01) with topsoil Na^+^, K^+^, Mg^2+^, and Ca^2+^ content (**Figure 10**). Brackish water irrigation decreased topsoil cation content and improved ion selectivity honeysuckle. This enhancement promoted intracellular K^+^ homeostasis and Na^+^ exclusion, thereby increasing the plant's K^+^/Na^+^ ratio (Liu et al., 2020). Under high salinity, plants maintain low cytosolic Na^+^ concentrations by limiting Na^+^ influx and enhancing efflux, preserving high K^+^/Na^+^ ratio essential for normal growth. Crucial roles in these processes are mediated by plasma membrane SOS1 Na^+^/H^+^ antiporter, high-affinity K^+^ transporters, and tonoplast exchangers Na^+^/H^+^ exchangers (Zhu, 2001; Almeida et al., 2017; Blumwald and Poole, 1985). Although constitutive SOS1/NHX activity in glycophytes is relatively low, NaCl stress induces expression. This induction facilitates vacuolar Na^+^ compartmentalization, reducing cytosolic toxicity and promoting growth (Mohammad, 2011). This suggests a potential three-tiered ion partitioning mechanism in honeysuckle. As irrigation increased, root Na^+^ content decreased markedly, whereas K^+^ content declined only slightly. Stem Na^+^ levels remained relatively stable, but K^+^ accumulation rose significantly. Concurrently, leaf Na^+^ content decreased, accompanied by an increase in K^+^. These observations reveal a coordinated three-tiered ion regulation strategy: selective root absorption (restricting Na^+^ uptake), stem-specific storage (Na^+^ translocation and sequestration), and leaf ion homeostasis maintenance (strict K^+^/Na^+^ regulation).

### 4.2 Effects of brackish water irrigation on oxidative damage and biomass in honeysuckle

Notably, MDA and H_2_O_2_ levels progress declined with increasing brackish water irrigation (**Figure 8**). As a reliable indicator of cellular stress severity, MDA reflects membrane lipid peroxidation (Peng et al., 2022). Contrary to studies showing elevated brackish water mineralization exacerbating salt stress and increasing these oxidative markers, our findings diverge from conventional patterns (Feng et al., 2024; Fal et al., 2022). The MDA content was significantly negatively correlated (*P* < 0.01) with the K^+^/Na^+^ ratio across all organs (**Figure 10**), aligning with ionic homeostasis correlations observed in salt-stressed cucumber (Coskun et al., 2016). The reduced oxidative damage in honeysuckle is mediated by: (i) enhanced Na^+^ leaching in surface soil alleviating salt stress; and (ii) activated antioxidant defense systems effectively scavenging excess ROS mitigating oxidative damage (Coskun et al., 2016).

In this study, base diameter, plant height, leaf area index and honeysuckle roots, stems, and leaf biomass increased significantly with irrigation volume (**Table 1**). This could be attributed to brackish water irrigation altering the ionic equilibrium between soil and plant, increasing the K^+^/Na^+^ ratio in plant tissues. Enhanced ratio improved water and nutrient uptake, promoting boosting biomass accumulation (Turcios et al., 2021; Zhang et al., 2017). K^+^/Na^+^ ratio positively correlated with biomass Maintaining elevated K^+^/Na^+^ ratio stimulates key plant growth and metabolism enzymes (Zhang et al., 2023), enhances photosynthetic efficiency (Munns, 2002), facilitate protein synthesis (Chen et al., 2013), and optimizes antioxidant system function (Chen et al., 2022b), collectively promoting plant growth. Biomass showed a significant positive correlation (*P* < 0.01) with the whole-plant Na^+^ accumulation in honeysuckle (**Figure 10**). With increased irrigation, the plant likely compartmentalizes Na^+^ in vacuoles to maintain cytosolic K^+^ homeostasis (Pan et al., 2016; Guo et al., 2022; Turcios et al., 2021; Yan et al., 2017). Therefore, the increased whole-plant Na^+^ accumulation should not be simply interpreted as "salt injury accumulation"; rather, it is a necessary byproduct of a Na^+^ inclusion strategy. This strategy allows honeysuckle to utilize the water and nutrients from brackish water irrigation to enhance biomass. The rise in biomass is thus a result of enhanced salt tolerance, and the concurrent increase in Na^+^ accumulation is an indicator of successful salt handling and growth.TF1 and TF2 increased significantly with brackish water irrigation compared to the non-irrigation control, likely due to elevated ATP production energizing root transporters (e.g., SOS1 Na^+^/H^+^ antiporters) for efficient Na^+^ exclusion or shoot translocation (Keisham et al., 2018; Karahara and Horie, 2021).

**Figure 11:**
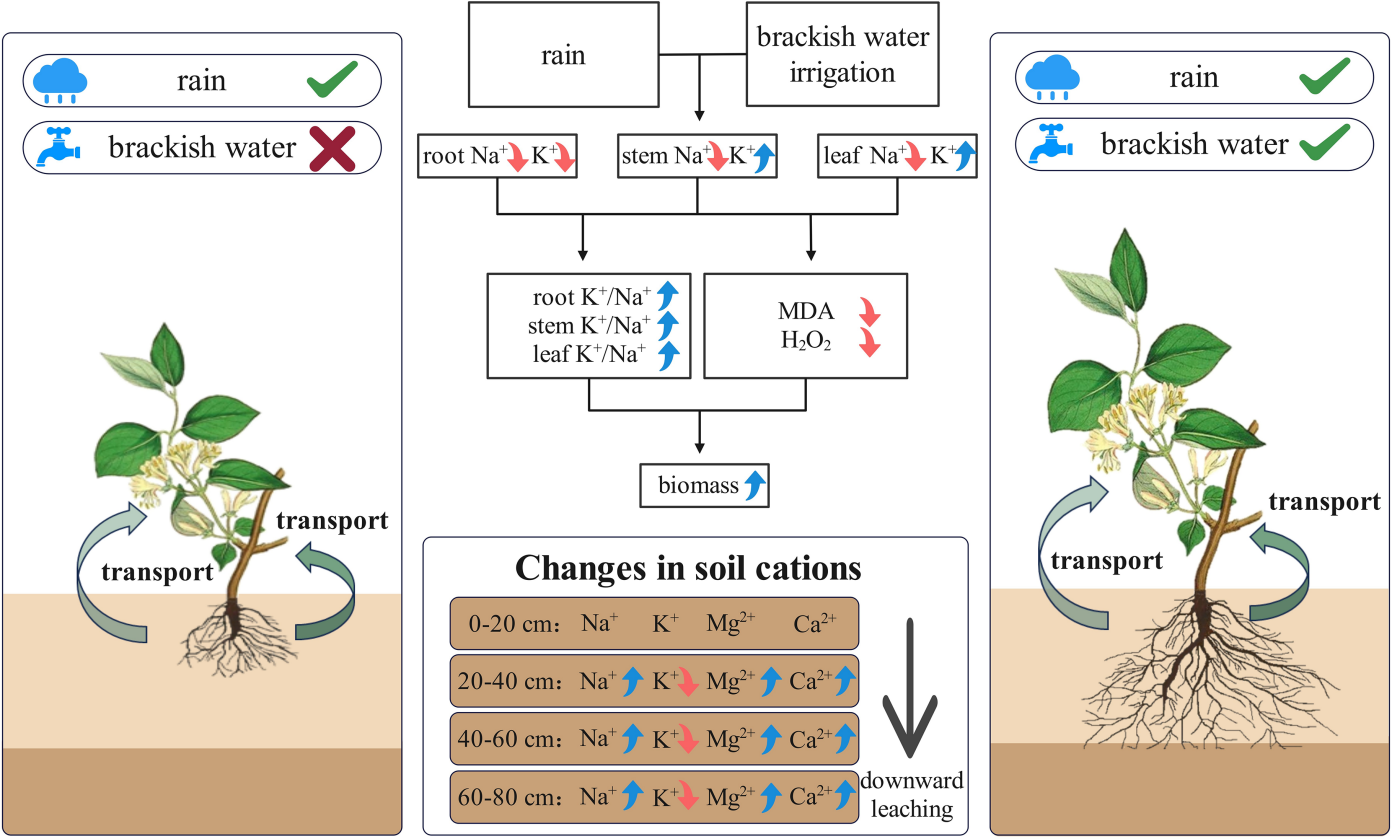
Schematic diagram of ion distribution and transport mechanisms in soil and honeysuckle under brackish water irrigation.

The conceptual diagram illustrates how precipitation and brackish water irrigation collectively reduce soil surface salinity, enhancing root development and aboveground biomass (**Figure 11**). This combined leaching effect substantially reduces surface salt accumulation, significantly decreasing Na^+^ in honeysuckle while maintaining ionic homeostasis. Consequently, elevated high K^+^/Na^+^ ratio reduces oxidative damage, and promotes plant growth. Brackish water irrigation facilitates deep salt ions leaching through the soil profile, causing substantial reductions in major cations (e.g., Na^+^, K^+^) in surface soil and optimizing salt distribution. Honeysuckle cultivation in the Yellow River Delta enhances adaptation to local climatic and brackish irrigation environments, maintaining high K^+^/Na^+^ ratio that reduces oxidative damage, promotes biomass accumulation, and improve saline soil restoration capacity. The synergistic mode alleviates agricultural water scarcity while enhancing saline-alkali soils remediation and productivity, establishing a pathway for coastal saline-alkali land ecological restoration.

### 4.3 Limitations and future research directions

This field cultivation study examined ionic homeostasis in coastal saline-alkali soils and basic growth responses of *Lonicera japonica* to brackish water irrigation. While generating significant findings, research limitations stem from field environmental variability, warranting further investigation:

1. Despite being supported by data from two consecutive growing seasons (2019-2020), the relatively short duration of our study presents a limitation. Soil salinity dynamics under brackish water irrigation are inherently long-term. Although we observed a consistent positive trend of leaching harmful ions from the topsoil, our two-year dataset may not fully encompass long-term risks. These include the potential accumulation of salts and specific ions (e.g., sodium, chloride) in deeper soil layers or groundwater, particularly over extended periods or under more extreme climatic conditions such as prolonged drought.
2. Effects on soil structure and microbial activity remain unexamined. These factors critically influence plant development through soil structure governs water retention and nutrient retention, and microbe-driven nutrient transformations. Future work should investigate how brackish irrigation alters coastal saline-alkali soil structure-microbial community dynamics and their interactive effects on plant growth and development.
3. Research should elucidate honeysuckle's physiological and molecular adaptations to saline irrigation, particular characterizing ion transport mechanisms, stress signaling pathways, and gene regulatory networks under salt stress conditions. Such insights will deepen mechanistic understanding of honeysuckle's salinity adaptation and inform salt-tolerant cultivar breeding.

Prolonged brackish water application may constrain system architecture, alter soil physicochemical properties, and suppress microbial functional activity (Arshad et al., 2022), ultimately counteracting coastal saline soil remediation efforts. Long-term use of brackish water for irrigation may saturate the buffering capacity of deep soil, forming a salt reservoir above the groundwater table (Yuan et al., 2019). Occasional heavy rainfall or extensive freshwater leaching could then displace these salts downward, eventually elevating groundwater salinity (Valenzuela et al., 2022). Additionally, salts leached into deeper layers may return to the root zone through groundwater rise or capillary action, posing a threat to crop growth (Li et al., 2024).

To mitigate these risks, we recommend: (1) Our study confirms the short-term efficacy of brackish water irrigation in reducing topsoil salinity, however, its long-term environmental impact remains uncertain. Given this uncertainty, future investigations must incorporate multi-annual monitoring. Such long-term data is critical to determine whether the observed desalination is stable or masks latent risks, such as the progressive accumulation of salts and specific ions (e.g., sodium) in the subsoil or groundwater. Consequently, sampling at key phenological stages (e.g., pre-planting, mid-growing, and post-harvest) over consecutive years is indispensable for resolving the dynamic interactions between irrigation, climate, and solute transport. (2) regular monitoring of soil solution electrical conductivity below 1 m depth; (3) alternating honeysuckle cultivation with deeper-rooted halophytes (e.g., *Salicornia europaea*) to transport excess salts back to the evaporation zone, maintaining long-term salt balance (Shrivastava and Kumar, 2015; Ma et al., 2013); and (4) installing shallow and deep drainage ditches under site-specific conditions to reduce salt accumulation.

## 5 Conclusion

Brackish water irrigation led to the leaching of Na^+^ and K^+^ from the topsoil, thereby reducing their content in the root zone. This process alleviated salt accumulation near the roots and resulted in a more favorable vertical salinity profile during the study period. It effectively regulated ion homeostasis in honeysuckle which significantly reduced Na^+^ concentration, increased K^+^ concentration and the K^+^/Na^+^ ratio, consequently enhanced biomass and total Na^+^ accumulation, thereby alleviating oxidative damage in the plant. These findings demonstrate honeysuckle's moderate salt tolerance and significant potential for ecological remediation in coastal saline-alkali soils. The study establishes a scientific basis for optimizing regional water resource and advancing sustainable agricultural in saline-alkali ecosystems.

## Data Availability

The raw data supporting the conclusions of this article will be made available by the authors, without undue reservation.
