# Supplementary material for: Brackish water irrigation boosts honeysuckle (Lonicera japonica Thunb.)-salt tolerance by regulating sodium partitioning and potassium homeostasis: implications for coastal saline soil
Source: Front Plant Sci. 2025 Dec 1;16:1655009. doi: 10.3389/fpls.2025.1655009 (PMC12702934; doi:10.3389/fpls.2025.1655009)
Supplement: Supplementary file 1 [file DataSheet1.docx]

Supplementary Material

# Supplementary Figure and Table

## **2.1 Supplementary Table**

Table S1: Results (F-values) of multifactorial analysis showing the effects of rainfall (R), treatment (T), year (Y), and their interactions on the contents of Na^+^, K^+^, Mg^2+^, and Ca^2+^ across different soil layers.

| Effect | 20 cm Na^+^ | 20 cm K^+^ | 20 cm Mg^2+^ | 20 cm Ca^2+^ | 40 cm Na^+^ | 40 cm K^+^ | 40 cm Mg^2+^ | 40 cm Ca^2+^ | 60 cm Na^+^ | 60 cm K^+^ | 60 cm Mg^2+^ | 60 cm Ca^2+^ | 80 cm Na^+^ | 80 cm K^+^ | 80 cm Mg^2+^ | 80 cm Ca^2+^ |
| --- | --- | --- | --- | --- | --- | --- | --- | --- | --- | --- | --- | --- | --- | --- | --- | --- |
| R | 392.51*** | 111.72*** | 427.45*** | 242.24*** | 31.16  *** | 23.5  *** | 213.94*** | 48.13  *** | 0.14 | 0.94 | 16.2  *** | 8.55  *** | 0.53 | 1.18 | 0.20 | 11.7  *** |
| T | 99.24  *** | 46.65  *** | 195.36*** | 155.09*** | 10.29  *** | 13.79  *** | 80.87  *** | 28.56  *** | 9.95  *** | 19.68  *** | 31.88  *** | 17.57  *** | 13.74  *** | 17.07  *** | 28.29  *** | 15.07  *** |
| Y | 74.09  *** | 0.01 | 112.91*** | 101.85*** | 18.01  *** | 10.44  ** | 13.38  *** | 0.75 | 33.53  *** | 32.45  *** | 13.32  *** | 0.02 | 45.92  *** | 2.12 | 21.57  *** | 0.11 |
| R × T | 9.79  *** | 3.59  ** | 2.37  * | 13.6  *** | 0.58 | 3.68  ** | 13.53  *** | 2.00  * | 0.72 | 2.56  * | 2.9  * | 3.68  ** | 1.10 | 4.97  *** | 9.1  *** | 2.41  * |
| R × Y | 166.58*** | 40.59  *** | 112.36*** | 52.52  *** | 8.45  *** | 7.06  ** | 89.41  *** | 12.24  *** | 9.54  *** | 28.64  *** | 8.21  *** | 12.91  *** | 2.07 | 21.61  *** | 4.97  ** | 0.613 |
| T × Y | 4.76  ** | 3.41  * | 2.53 | 9.31  *** | 6.70  *** | 8.41  *** | 10.6  *** | 13.04  *** | 1.35 | 7.28  *** | 3.45  * | 6.53  *** | 2.52 | 3.07  * | 5.95  * | 9.31  *** |
| R×T×Y | 17.28  *** | 4.62  *** | 29.47  *** | 25.16  *** | 2.00 | 1.36 | 3.22  ** | 1.01 | 2.41  * | 2.76  * | 2.10 | 2.87  * | 1.48 | 6.06  *** | 12.87  *** | 1.97 |

*, ** and *** represent signiﬁcant differences at *P* < 0.05, *P* < 0.01 and *P* < 0.001, respectively. The data for K^+^, Mg^2+^, and Ca^2+^ at 20 cm, Ca^2+^ at 40 cm, K^+^ and Mg^2+^ at 60 cm, and K^+^ and Mg^2+^ at 80 cm were subjected to logarithmic transformation.

## **2.2 Supplementary Figure**


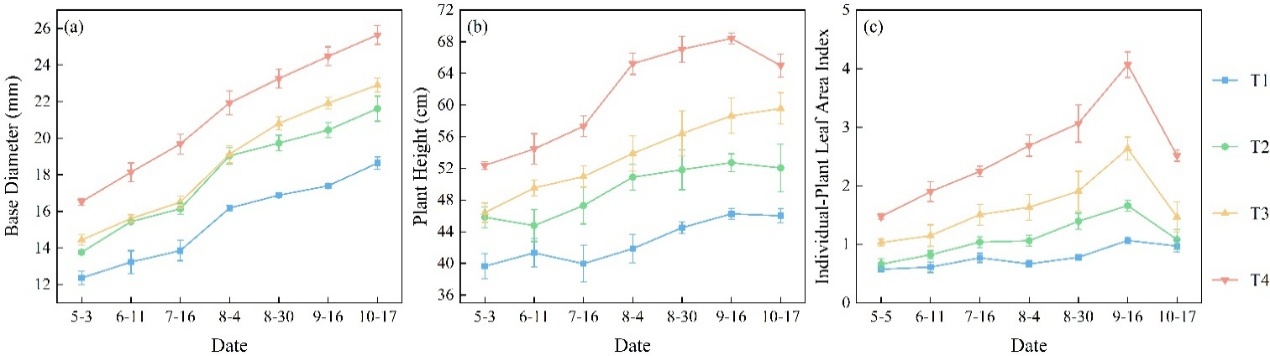


Figure S1: Seasonal dynamics of leaf area index, basal diameter, and plant height of honeysuckle under different brackish water irrigation levels in 2020 (mean ± SE, n＝4).
